# Outcomes of pulmonary metastasectomy in urological cancer: insights from a retrospective cohort

**DOI:** 10.7717/peerj.21171

**Published:** 2026-05-04

**Authors:** Ryusei Yoshino, Kengo Takahashi, Nozomi Hatanaka, Akane Ito, Nanami Ujiie, Shunsuke Yasuda, Masahiro Kitada

**Affiliations:** Thoracic Surgery, Asahikawa Medical University, Asahikawa, Japan

**Keywords:** Pulmonary metastasectomy, Urological malignancies, Renal cell carcinoma, Oligometastatic disease, Surgical outcome

## Abstract

Pulmonary metastasectomy has been established as a treatment option for select patients with metastatic disease. However, its role in urological malignancies remains underexplored. This study aimed to evaluate the clinical outcomes of pulmonary metastasectomy in patients with urological cancers, with a focus on renal cell carcinoma (RCC). We retrospectively reviewed 18 patients who underwent pulmonary resection for suspected metastases from urological malignancies at a single institution between 2014 and 2023. A subgroup analysis of 11 patients with RCC was conducted. The cohort included 13 patients with kidney cancer, four with testicular tumors, and one with prostate cancer. Pulmonary lesions were confirmed to be metastatic in 14 patients and inflammatory changes in four. The median disease-free interval was 25 months and the median overall survival after pulmonary resection was 113 months. In the RCC subgroup, the 10-year survival rate exceeded 50%, despite a 66.7% recurrence rate. No significant prognostic factors were identified, although favorable trends were observed for younger age, solitary metastases, and absence of vascular invasion. In urological malignancies, particularly renal cell carcinoma, pulmonary metastasectomy was associated with prolonged survival in carefully selected patients. Surgical resection may serve both therapeutic and diagnostic purposes, supporting its role within a multidisciplinary treatment strategy.

## Introduction

Pulmonary metastases represent a common clinical scenario in the course of various malignancies, and surgical resection—pulmonary metastasectomy—has been performed in select patients for decades to achieve disease control or a potential cure (Raveglia et al., 2021; Meacci et al., 2017). While most literature on metastasectomy focuses on sarcomas, colorectal cancer, and renal cell carcinoma (RCC), pulmonary metastases from urological malignancies remain relatively under-investigated as a distinct clinical entity. A multi-institutional study of urogenital tumors identified 22 of 126 metastasectomy cases as originating from urological cancers, highlighting the rarity and diagnostic challenges of these lesions (Ceylan, Batı han & Kaya, 2023).

RCC is known for its unpredictable clinical course, with oligometastatic pulmonary disease occurring in a notable subset. Testicular germ cell tumors are highly chemosensitive; however, residual pulmonary nodules frequently require surgical resection after first-line chemotherapy (Caso et al., 2021). In contrast, pulmonary metastases from urothelial carcinoma (UC) or prostate cancer remain rare; however, recent data suggest that UC metastasectomy may prolong survival in select patients (Yamauchi et al., 2024).

Advances in systemic therapies, including immune checkpoint inhibitors (ICIs), vascular endothelial growth factor-targeted agents, and novel chemotherapeutic regimens, have significantly improved the survival of patients with metastatic urological cancers (Tung & Sahu, 2021). Nevertheless, the optimal integration of local therapies, such as metastasectomy, into modern multimodal treatment paradigms remains unresolved. The increasing use of imaging surveillance in urological oncology has led to the more frequent detection of small, asymptomatic pulmonary nodules, which may represent true metastases, benign lesions, or residual post-treatment inflammation. In this context, metastasectomy serves not only as a therapeutic intervention but also as a tool for histological confirmation, prognostication, and treatment planning.

Despite these clinical imperatives, data on outcomes, recurrence patterns, and long-term prognosis following pulmonary resection in urological primary tumors remain limited. To address this knowledge gap, we retrospectively reviewed 18 patients with suspected pulmonary metastases from urological cancers, focusing on clinical characteristics, surgical outcomes, and long-term survival, with particular emphasis on RCC.

## Materials and Methods

### Study design and patient selection

This retrospective, single-institution study was conducted at our institution. The need for informed consent was waived owing to the retrospective nature of the study, with an opt-out procedure available on the hospital website in accordance with the Declaration of Helsinki. We reviewed the medical records of the patients who underwent pulmonary resection for suspected metastases originating from urological malignancies between April 2014 and December 2023. The inclusion criteria were as follows: (1) history of primary urological cancer (kidney, prostate, or testis), (2) presence of pulmonary nodules clinically suspected as metastases, and (3) having undergone curative-intent pulmonary resection. Patients with incomplete clinical records or non-urological primary tumors were excluded.

### Data collection

Clinical data were extracted from the electronic medical records, surgical reports, and pathology archives. Data on the following variables were collected:

• Patient demographics: age at primary surgery and metastasectomy and sex.
• Primary tumor characteristics: anatomical site, histologic type, pathological Tumor Node Metastasis stage (Union for International Cancer Control), tumor grade (for RCC), infiltrative growth (INF) pattern (a–c), vascular and lymphatic invasion, and surgical margin status (Patriarca, Ferretti & Zanetti, 2017; Bertero et al., 2018).
• Pulmonary metastasis characteristics: number, size, laterality, surgical method, resection completeness, and histological classification (true metastasis *vs.* inflammatory changes).
• Pulmonary function data: data on respiratory function parameters, including vital capacity (VC), forced expiratory volume in 1 s (FEV1), percent of predicted vital capacity (%VC), the FEV1 to forced vital capacity ratio (FEV1%), and classification of ventilatory impairment.
• Treatment course and outcomes: adjuvant therapy, recurrence, disease-free interval (DFI), overall survival (OS), and follow-up status.

### Surgical procedures

All surgeries were performed *via* video-assisted thoracoscopic surgery (VATS), hybrid VATS, or robotic-assisted thoracic surgery (RATS) based on the lesion location, tumor burden, and surgeon discretion. Wedge resection was the standard approach, and lobectomy was performed when warranted based on the tumor location or prior interventions. Resection was considered complete (R0) if both macroscopic and microscopic margins were negative. Operative time and intraoperative bleeding volume were recorded for all patients.

### Histopathological evaluation

All surgical specimens were reviewed by board-certified pathologists specializing in thoracic and genitourinary pathology. Resected nodules were categorized as either viable metastatic carcinoma (based on morphological and immunohistochemical features consistent with the primary tumor) or nonviable/treatment-related lesions (*e.g.*, fibrosis, necrosis, and granulomatous inflammation). RCC tumors were graded using the World Health Organization/International Society of Urological Pathology system (Patriarca, Ferretti & Zanetti, 2017; Bertero et al., 2018). Pathological features, such as INF pattern and vascular/lymphatic invasion, were recorded.

### Follow-up protocol

Postoperative follow-up was conducted every 3–6 months for the first 2 years and every 6–12 months thereafter. Follow-up evaluations included chest computed tomography (CT), abdominal imaging (CT or ultrasonography), and serum tumor markers, when appropriate. Recurrence was confirmed using imaging or histopathology, when available. Maintenance therapy was defined as any chemotherapy, targeted therapy, or immune checkpoint inhibitor administered before or after pulmonary metastasectomy. In patients with stage IV disease, postoperative treatment was classified as maintenance or salvage therapy rather than adjuvant therapy.

### Subgroup analysis of patients with RCC

Given the predominance of patients with RCC in this cohort, a dedicated subgroup analysis was conducted. The variables examined included tumor grade, pathological stage, INF classification, vascular and lymphatic invasion, and pulmonary metastasis parameters (number, size, and laterality). Outcomes, such as recurrence, DFI, and OS were analyzed according to these clinicopathological features.

### Statistical analysis

Categorical variables were summarized as frequencies and percentages and continuous variables as medians with ranges. Survival curves were estimated using the Kaplan–Meier method, and group comparisons were performed using the log-rank test. The DFI was defined as the time from urological cancer resection to the diagnosis of metastatic lung tumors after lung resection. OS was defined as the duration from urological cancer resection to death or last follow-up. A *p*-value < 0.05 was considered statistically significant. All analyses were performed using GraphPad Prism 10 (GraphPad Software Inc., La Jolla, CA, USA).

### Handling of missing data

Patients with incomplete key clinical data (*e.g.*, lack of pathological confirmation or unknown surgical outcomes) were excluded at the time of the initial screening. Minor missing data points (*e.g.*, pulmonary function variables in 1–2 patients) were treated as missing completely at random and excluded from the relevant analysis without imputation.

### Ethical considerations

This study was approved by the Institutional Review Board of Asahikawa Medical University (Approval no. 25021). Informed consent was waived due to the retrospective study design.

## Results

### Patient characteristics

Eighteen patients with suspected pulmonary metastases originating from urological malignancies were included (Table 1). Their median age at the time of primary surgery was 62 (range, 24–84) years, and a male predominance was noted (15 male, 83.3%; three female, 16.7%). The primary tumor sites were the kidneys (*n* = 13, 72.2%), testes (*n* = 4, 22.2%), and prostate (*n* = 1, 5.6%). Among kidney tumors, clear cell RCC was the most frequent histological subtype (*n* = 11, 84.6%), followed by UC of the renal pelvis (*n* = 2, 15.4%). In one patient with prostate cancer, the histological type was unknown. Testicular tumors included mucinous adenocarcinoma (*n* = 1), choriocarcinoma (*n* = 1), non-seminomatous germ cell carcinoma (*n* = 1), and mixed germ cell tumors (*n* = 1). The median size of the primary tumor was 65 (range, 0–160) mm. The pathological stage (pStage) distribution was as follows: stage 0, *n* = 1, 5.6%; stage I, *n* = 6, 33.3%; stage II, *n* = 1, 5.6%; stage III, *n* = 8, 44.4%; and unknown stage, *n* = 2, 11.1%. Regarding initial treatment, 14 patients (77.8%) underwent surgery alone, three (16.7%) received adjuvant chemotherapy, and one (5.6%) received adjuvant radiation therapy.

**Table 1:** Characteristics of patients with primary urological cancers (*n* = 18).

Characteristic		
Age (years)		
Median	62	
Range	24–84	
Sex		
Male	15	83.3%
Female	3	16.7%
Primary location		
Kidney	13	72.2%
Prostate	1	5.6%
Testis	4	22.2%
Histological type (kidney)		
Clear cell RCC	11	84.6%
Urothelial carcinoma of renal pelvis	2	15.4%
Histological type (prostate)		
Unknown	1	100.0%
Histological type (testis)		
Mucinous adenocarcinoma	1	25.0%
Choriocarcinoma	1	25.0%
Non-seminomatous germ cell carcinoma	1	25.0%
Mixed germ cell tumor	1	25.0%
Tumor size (mm)		
Median	65	
Range	0–160	
pStage		
0	1	5.6%
1	6	33.3%
2	1	5.6%
3	8	44.4%
4	0	0.0%
Unknown	2	11.1%
Treatment		
Surgery alone	14	11.9%
Chemotherapy after surgery	3	16.7%
Radiation therapy after surgery	1	5.6%

**Notes.**

RCC renal cell carcinoma

### Characteristics of pulmonary metastases

At the time of pulmonary metastasectomy, the median patient age was 67 (range, 24–85) years (Table 2). The number of pulmonary lesions was one in 12 patients (66.7%), 2–9 in three patients (16.7%), and ≥10 in three patients (16.7%). The median size of the metastatic tumors was 10 (range, 0–25) mm. Most metastases were unilateral (*n* = 16, 88.9%); bilateral involvement was noted in two patients (11.1%). Seventeen patients (94.4%) underwent partial lung resection, and one (5.6%) underwent lobectomy. Segmentectomy was not performed. Complete resection was achieved in all 18 patients. Histopathological analysis confirmed true metastatic carcinoma in 14 patients (77.8%), whereas four lesions (22.2%) represented treated metastases or inflammatory changes. Following surgery, 12 patients (66.7%) received adjuvant chemotherapy, whereas six (33.3%) did not receive further therapy. Recurrence occurred in nine patients (50.0%), and the median DFI was 25 (range, 3–204) months. At the time of analysis, 11 patients (61.1%) were alive, three (16.7%) had died, and four (22.2%) were censored. The median OS after pulmonary metastasectomy was 113 (range, 28–223) months.

**Table 2:** Characteristics of metastatic lesions (*n* = 18).

Characteristic		
Age (years)		
Median	67	
Range	24–85	
Number of tumors		
1	12	66.7%
2–9	3	16.7%
≥10	3	16.7%
Tumor size (mm)		
Median	10	
Range	0–25	
Laterality		
Unilateral	16	88.9%
Bilateral	2	11.1%
Surgical resection		
Partial resection	17	94.4%
Segmentectomy	0	0.0%
Lobectomy	1	5.6%
Outcome of metastasectomy		
Complete	18	100.0%
Incomplete	0	0.0%
Other metastasis		
Positive	2	11.1%
Negative	16	88.9%
Histological type		
Metastatic carcinoma	14	77.8%
Treated metastases	4	22.2%
Maintenance therapy after surgery		
None	6	33.3%
Chemotherapy	12	66.7%
Recurrence after metastasectomy		
Negative	9	50.0%
Positive	9	50.0%
DFI (months)		
Median	25	
Range	3–204	
Outcome		
Alive	11	61.1%
Dead	3	16.7%
Censored	4	22.2%
OS after metastasectomy (months)		
Median	113	
Range	28–223	

**Notes.**

DFI disease-free interval OS overall survival

### Surgical characteristics

Pulmonary function testing revealed a mean VC of 3,125 (range, 1,400–4,760) mL, and a mean %VC of 99% (range, 65.6–123.2%; Table 3). The mean FEV1 was 2,447 (range, 929–3,740) mL, with a mean FEV1% of 81% (range, 63.7–105.9%). Preoperative respiratory function was normal in 13 patients (72.2%), whereas three (16.7%), one (5.6%), and one (5.6%) had restrictive, obstructive, and mixed impairment, respectively. The mean operative time was 66 (range, 32–308) min, and intraoperative bleeding was minimal (mean, 3 mL; range, 0–20 mL). Surgical approaches included complete VATS, hybrid VATS, and RATS in seven (38.9%), 10 (55.6%), and one patient (5.6%), respectively. Maintenance therapies were administered in a heterogeneous manner, reflecting real-world clinical practice. Among patients with renal cell carcinoma, targeted therapies and immune checkpoint inhibitors were frequently used, including pazopanib, axitinib, cabozantinib, nivolumab, and pembrolizumab-based combinations. Some patients received systemic therapy before pulmonary metastasectomy, whereas others were treated postoperatively as maintenance or salvage therapy. The timing and regimen of systemic therapy varied across patients, and no uniform treatment protocol was applied.

**Table 3:** Surgical characteristics (*n* = 18).

Characteristic		
VC (mL)		
Mean	3,125	
Range	1,400–4,760	
%VC (%)		
Mean	99.0	
Range	65.6–123.2	
FEV1 (mL)		
Mean	2,447	
Range	929–3,740	
FEV1% (%)		
Mean	81.0	
Range	63.7–105.9	
Disease		
Normal	13	72.2%
Restrictive ventilatory impairment	3	16.7%
Obstructive ventilatory impairment	1	5.6%
Restrictive + obstructive ventilatory impairment	1	5.6%
Surgery time (min)		
Mean	66	
Range	32–308	
Bleeding (mL)		
Mean	3	
Range	0–20	
Surgical method		
Complete VATS	7	38.9%
Hybrid VATS	10	55.6%
RATS	1	5.6%

**Notes.**

VC vital capacity %VC percent of predicted vital capacity FEV1 forced expiratory volume in 1 s FEV1% percent of forced expiratory volume in 1 s VATS video-assisted thoracoscopic surgery RATS robotic-assisted thoracic surgery

### Subgroup analysis: patients with carcinoma of renal origin (*n* = 12)

Detailed clinical and pathological characteristics of the 12 patients with carcinoma of renal origin are shown in Table 4. Their median age at nephrectomy was 65 (range, 51–84) years and that at pulmonary resection was 68 (range, 51–85) years. The tumor grade distributions were as follows: grade 1, *n* = 5, 41.7%; grade 2, *n* = 6, 50.0%; and grade 3, *n* = 1, 8.3%. Vascular invasion was present in five patients (41.7%) and the INF classification was INFa and INFb in 11 and one patient, respectively. All patients had negative surgical margins. During pulmonary resection, 11 patients (91.7%) underwent partial resection and one (8.3%) underwent lobectomy. Bilateral metastases were present in one patient (8.3%), and complete resection was achieved in all patients. Other metastases outside the lungs were present in one patient (8.3%). Adjuvant chemotherapy was administered to four patients (33.3%), whereas eight (66.7%) received no further treatment. Recurrence occurred in eight patients (66.7%), with a median DFI of 30 (range, 3–204) months. At the final follow-up, six patients (50.0%) were alive, three (25.0%) had died, and three (25.0%) were censored.

**Table 4:** Characteristics of patients with primary cancer of the kidney (*n* = 12).

Characteristic						
Age at nephrectomy (years)				Age at PM (years)		
Median	65			Median	68	
Range	51–84			Range	51–85	
Sex				Number of tumors		
Male	9	75.0%		1	8	66.7%
Female	3	25.0%		2–9	1	8.3%
Tumor size (mm)				≥10	2	16.7%
Median	65			Tumor size (mm)		
Range	0–160			Median	10	
Grade				Range	5–25	
1	5	41.7%		Laterality		
2	6	50.0%		Unilateral	11	91.7%
3	1	8.3%		Bilateral	1	8.3%
INF classification				Surgical resection		
a	11	91.7%		Partial resection	11	91.7%
b	1	8.3%		Segmentectomy	0	0.0%
c	0	0.0%		Lobectomy	1	8.3%
Vascular invasion				Outcome of PM		
Negative	7	58.3%		Complete	12	100.0%
Positive	5	41.7%		Incomplete	0	0.0%
Lymphatic invasion				Other metastasis		
Negative	12	100.0%		Negative	11	91.7%
Positive	0	0.0%		Positive	1	8.3%
Surgical margin				Maintenance therapy after PM		
Negative	12	100.0%		None	8	66.7%
Positive	0	0.0%		Chemotherapy	4	33.3%
pStage				Recurrence after PM		
0	1	8.3%		Negative	4	33.3%
1	6	50.0%		Positive	8	66.7%
2	1	8.3%		DFI (months)		
3	4	33.3%		Median	30	
Treatment				Range	3–204	
Surgery alone	12	100.0%		Outcome		
				Alive	6	50.0%
				Dead	3	25.0%
				Censored	3	25.0%

**Notes.**

INF infiltrative growth PM pulmonary metastasis DFI disease-free interval

### Univariate analysis of patients with RCC

**Figure 1:**
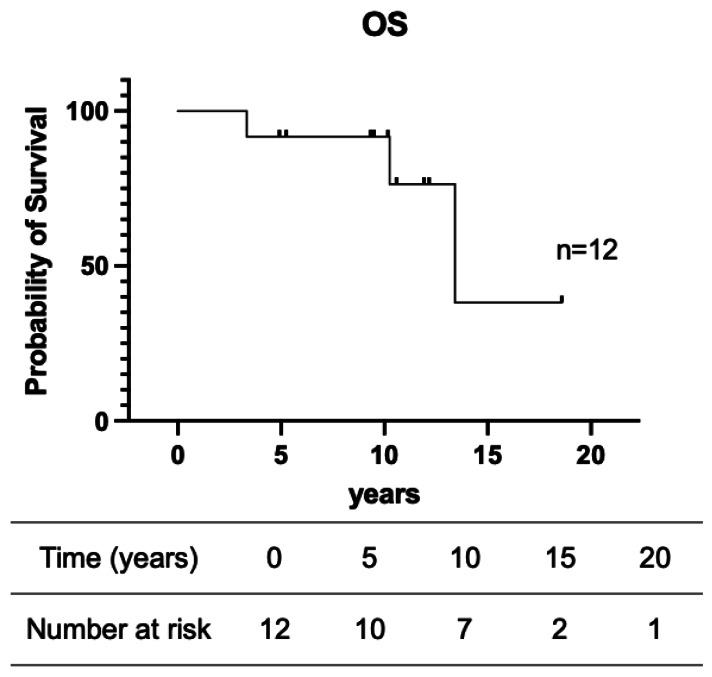
Survival outcomes of patients with renal-origin pulmonary metastases. OS, overall survival.

The results of univariate analysis of the prognostic factors for patients with RCC are summarized in Table 5. Although no significant associations were found, several trends were observed. Patients aged <65 years had a 10-year survival rate of 100%, compared with 83.3% for those aged ≥65 years (*p* = 0.5637). Patients with a primary tumor size <60 mm showed 5- and 10-year survival rates of 80.0%. Patients with grade 1 tumors had a 100% survival rate, whereas those with grade 2 or 3 tumors showed a slightly lower survival rate (85.7%; *p* = 0.7675). Patients without vascular invasion had a 100% 5-year survival rate, compared with 80.0% in those with vascular invasion (*p* = 0.3752). pStage did not significantly affect outcomes (*p* = 0.2489). Similarly, the number and size of metastatic tumors were not significant factors, although all patients with solitary or small (<10 mm) metastases achieved a 100% 10-year survival rate. The 5-, 10- and 15-year survival rates were 91.7%, 91.7%, and 38.2%, respectively (Fig. 1). Multivariate Cox regression analysis was attempted; however, it could not be reliably performed because of the limited number of events relative to the number of candidate variables, resulting in model instability.

**Table 5:** Univariate analysis of prognostic factors for patients with renal cell carcinoma.

Variable (primary cancer of the kidney)	5-year survival (%)	10-year survival (%)	*p*-value
Age at primary surgery (years)			0.5637
<65 (*n* = 6)	100.0	100.0	
≥65 (*n* = 6)	83.3	83.3	
Primary tumor size (mm)			0.5827
<60 (*n* = 5)	80.0	80.0	
≥60 (*n* = 7)	100.0	100.0	
Grade			0.7675
1 (*n* = 5)	100.0	100.0	
2 or 3 (*n* = 7)	85.7	85.7	
Vascular invasion			0.3752
Positive (*n* = 5)	80.0	80.0	
Negative (*n* = 7)	100.0	100.0	
pStage			0.2489
0 or 1 (*n* = 7)	100.0	100.0	
2 or 3 (*n* = 5)	100.0	80.0	
Number of metastatic tumors			0.7055
<4 (*n* = 9)	100.0	88.9	
≥4 (*n* = 3)	100.0	100.0	
Size of metastatic tumors (mm)			0.2568
<10 (*n* = 3)	100.0	100.0	
≥10 (*n* = 9)	100.0	88.9	

## Discussion

In this retrospective cohort of 18 patients with suspected pulmonary metastases from urological malignancies, pulmonary metastasectomy achieved a median OS of approximately 9.4 years, with >60% surviving for 10 years. In the RCC patient subgroup, even those with recurrence post-resection demonstrated extended survival, supporting the potential role of pulmonary metastasectomy in carefully selected patients.

Our findings of durable OS after pulmonary metastasectomy complement emerging evidence from large cohort studies and meta-analyses. A Japanese multi-institutional study of 100 patients with UC reported 5-year OS and disease-free survival rates of 59% and 46%, respectively, with larger lesion size and synchronous metastases identified as poor prognostic factors (Yamauchi et al., 2024). Similarly, a 2024 World Journal of Urology study of non-clear cell RCC showed that metastasectomy combined with nephrectomy significantly improved the 5-year OS (67.3 *vs.* 24.0 months), particularly in patients with metachronous or non-hepatic metastases (Dai et al., 2024). Reports on pulmonary metastasectomy in urological cancers demonstrated 5-year survival rates of 45%–75%, with tumor size and metastatic burden being significant prognostic factors (Muilwijk et al., 2020). The >50% 10-year survival rate observed in our RCC subgroup further suggests that meticulous patient selection, based on small lesion size, metachronous oligometastases, and absence of nodal disease, may be critical for achieving long-term disease control.

Beyond tumor clearance, resection of residual pulmonary lesions after systemic therapy offers diagnostic refinement and therapeutic adaptability. In non-small cell lung cancer, circulating tumor DNA is emerging as a robust biomarker, with postoperative detection of minimal residual disease predicting recurrence significantly earlier, compared with imaging (Wang et al., 2025; Tian et al., 2024). Similar trends have been observed in urological cancers; meta-analyses in bladder cancer and early-phase studies in RCC indicate that circulating tumor DNA can guide adjuvant therapy decisions (Katsimperis et al., 2025). Surgical resection in this context provides histological confirmation, distinguishes viable tumors from treatment-induced inflammation or fibrosis, and serves as a pivotal point for therapy escalation or de-escalation. Moreover, the resected specimens enable spatial immunoprofiling and genomic sequencing, offering insights into resistance mechanisms and informing future therapeutic strategies. Our findings, in which resection clarified the ambiguous radiological findings and informed systemic therapy decisions, align with this evolving paradigm.

Recent therapeutic advances, particularly introduction of ICIs, have significantly improved outcomes in metastatic RCC and UC (Kato & Uchida, 2023). However, systemic therapy alone may not achieve complete eradication of residual disease in all patients. Multidisciplinary management models advocate a combined approach: systemic therapy to induce regression, followed by local therapy to eradicate resistant or indolent lesions (Dason et al., 2023). Several prospective studies and international guidelines support pulmonary metastasectomy in carefully selected patients with oligometastases, especially in those showing a favorable response to systemic therapy (Berghen et al., 2020). Our results reinforce this shift toward multimodal care, demonstrating the relevance of pulmonary resection in the modern therapeutic era.

This study also clarifies the evolving role of surgery beyond cytoreduction. The biological rationale for metastasectomy is supported by the concept of oligometastatic disease, a state where limited metastatic potential allows long-term control through localized interventions (Hafez & Gettinger, 2020). Surgical debulking in this setting may reduce tumor load to below immune escape thresholds, enhancing systemic immune surveillance and synergizing with ICIs (Goswami et al., 2025). In select patients, metastasectomy may improve psychological well-being and quality of life by reducing uncertainty and limiting the toxicity burden associated with prolonged systemic therapy.

To place our findings in the context of existing evidence, we summarized previously published outcomes of pulmonary metastasectomy for urological malignancies in PubMed-indexed studies (Table 6). Across these heterogeneous retrospective cohorts, long-term survival after pulmonary metastasectomy has been consistently reported only in highly selected patients, typically characterized by limited metastatic burden, small tumor size, long disease-free intervals, and complete resection. In urothelial carcinoma and renal cell carcinoma, reported 5-year overall survival rates generally range from approximately 45% to 75%, particularly among patients with solitary or oligometastatic pulmonary lesions. Importantly, most prior studies lack nonsurgical comparator groups and reflect substantial variability in systemic therapy, underscoring the inherent selection bias shared by the present referral-based surgical cohort. In this context, our results should be interpreted not as evidence of a causal survival benefit of surgery, but as real-world outcomes observed in carefully selected patients, which are broadly consistent with those reported in the existing literature.

**Table 6:** Summary of published outcomes of pulmonary metastasectomy for urological malignancies in PubMed-indexed studies.

Primary cancer	Study (year)	Study design	Number	Pulmonary metastatic burden	Systemic therapy context	Survival outcomes	Reported adverse prognostic factors
UC	Yamauchi et al. (2024)	Multicenter retrospective (Japan)	100	Mean max diameter 21 ± 15 mm; lung-only or lung-dominant	Pre-/early-ICI era; heterogeneous perioperative therapy	5-yr OS 59%; 5-yr DFS 46%	Larger tumor size; distant metastases at primary treatment
UC	Muilwijk et al. (2020)	Single-center retrospective	22	Solitary (*n* = 8) *vs* multiple (*n* = 5); size cut-off 8 mm	Platinum-based chemotherapy ± surgery	Lung metastasectomy: 5-yr OS 55.9%; solitary lesions: 87.5%	>1 pulmonary lesion; lesion size >8 mm; UTUC involvement
RCC	Raveglia et al. (2021)	Multicenter retrospective	210	Mean 2.1 lesions (range 1–13); mean diameter 2.1 cm; unilateral 59%	Targeted therapy era; limited ICI use	5-yr OS 60%; 10-yr OS 34%	Synchronous metastases; low performance status
RCC	Meacci et al. (2017)	Single-center retrospective	27	Solitary *vs* multiple; diameter ≥2 cm analyzed	Pre-ICI era	5-yr OS 75%; 10-yr OS 59%	Tumor diameter ≥2 cm; short DFI
RCC	Zhao et al. (2017)	Systematic review & meta-analysis	1,447	Heterogeneous definitions across studies	Pre-ICI era	Pooled OS: 1-yr 84%; 5-yr 43%; 10-yr 20%	Short DFI; incomplete resection; multiple metastases; large tumor size
GCT	Pfannschmidt et al. (2006)	Post-chemotherapy thoracic metastasectomy	52	Residual pulmonary lesions after chemotherapy	Cisplatin-based chemotherapy	5-yr survival 75.8%	Elevated tumor markers; incomplete resection
Mixed urogenital	Ceylan, Batıhan & Kaya (2023)	Single-center retrospective	22	Mixed burden; lung-predominant metastases	Heterogeneous	Survival benefit suggested in selected patients	Multiple metastases; tumor size >3 cm; short DFI
Mixed urogenital	Radulescu et al. (2014)	Retrospective institutional experience	26	Lung-dominant; limited number of lesions	Heterogeneous	Short-term survival reported	Multiple lesions; bilateral disease

**Notes.**

UC urothelial carcinoma RCC renal cell carcinoma GCT germ cell tumor ICI immune checkpoint inhibitor OS overall survival DFS disease-free survival UTUC upper tract urothelial carcinoma

Nonetheless, our study has limitations. The small sample size and retrospective design introduce potential selection and information biases. The heterogeneity in primary tumor histology and systemic treatment strategies limits the generalizability of our findings. Moreover, the lack of a nonsurgical comparator group precludes definitive conclusions regarding the survival benefit of metastasectomy. The incremental value of pulmonary resection in this era of effective systemic therapies, including ICI-based combinations, warrants further investigation. Future prospective, biomarker-integrated, multi-institutional studies are needed to determine the optimal timing, indications, and long-term benefits of pulmonary metastasectomy in patients with urological malignancies.

## Conclusions

In this retrospective single-institution cohort, pulmonary metastasectomy for urological malignancies, particularly renal cell carcinoma, was associated with long-term survival in carefully selected patients. However, given the limited sample size and heterogeneity of systemic treatment strategies, these findings should be interpreted cautiously and are not intended to establish definitive treatment recommendations.

Beyond its potential therapeutic role, pulmonary resection provided important diagnostic information and contributed to individualized treatment decision-making in the context of contemporary systemic therapies. Our results support the role of pulmonary metastasectomy as a component of multidisciplinary management in selected cases and highlight the need for larger, prospective, multi-institutional studies to better define patient selection criteria and optimal integration of metastasectomy into modern treatment algorithms.

## Supplemental Information

10.7717/peerj.21171/supp-1 Supplemental Information 1 Raw data

10.7717/peerj.21171/supp-2 Supplemental Information 2 STROBE checklist

## Abbreviations

RCC: renal cell carcinoma
UC: urothelial carcinoma
ICIs: immune checkpoint inhibitors
INF: infiltrative growth pattern
VC: vital capacity
FEV1: forced expiratory volume in 1 s
%VC: percent predicted vital capacity
FEV1%: FEV1/FVC ratio (percent)
DFI: disease-free interval
OS: overall survival
VATS: video-assisted thoracoscopic surgery
RATS: robotic-assisted thoracic surgery
R0: complete (margin-negative) resection
CT: computed tomography
pStage: pathological stage

## Additional Information and Declarations

### Data Availability

The following information was supplied regarding data availability: The raw data is available in the Supplemental File.
